# Cloning of a *HcCreb* gene and analysis of its effects on nacre color and melanin synthesis in *Hyriopsis cumingii*

**DOI:** 10.1371/journal.pone.0251452

**Published:** 2021-05-20

**Authors:** Mengying Zhang, Xiajun Chen, Jinpan Zhang, Jiale Li, Zhiyi Bai

**Affiliations:** 1 Key Laboratory of Freshwater Aquatic Genetic Resources, Ministry of Agriculture, Shanghai Ocean University, Shanghai Ocean University, Shanghai, China; 2 Shanghai Collaborative Innovation for Aquatic Animal Genetics and Breeding, Shanghai Ocean University, Shanghai, China; 3 Fisher Institute of Anhui Academy of Agricultural Sciences, Hefei, China; 4 Shanghai Engineering Research Center of Aquaculture, Shanghai Ocean University, Shanghai, China; Universiti Sains Malaysia, MALAYSIA

## Abstract

Creb (Cyclic AMP response element binding protein) is a nuclear regulatory factor that regulates transcription through autophosphorylation. In melanocytes, cAMP’s corresponding elements bind to the Creb protein to autophosphorylation and activate MITF (Microphthalmia-associated transcription factor). MITF stimulates Tyrosine(tyr) to induce melanocytes to differentiate into eumelanin and pheomelanin. In this study, a *HcCreb* gene in *Hyriopsis cumingii* was cloned and its effects on melanin synthesis and nacre color were studied. *HcCreb* was expressed in both purple and white mussels, and there was a significant difference in expression between adductor muscle (*p*<0.01) and mantle tissue (*p*<0.05). Other tissues did not show significant differences (except for gill tissue), and in general, the level of mRNA expression was higher in purple mussels than in white mussels. In both white and purple mussels expression levels in gill tissue was the highest, followed by the mantle. Strong and specific mRNA signals were detected in the dorsal epithelial cells of the mantle pallial layer, indicating that *HcCreb* may be involved in nacre formation. After arbutin treatment, the expression of *HcCreb* decreased significantly. By further testing the changes in mantle melanin content it was found that the melanin content after arbutin treatment decreased significantly compared to the control group (*p<*0.05). It is speculated that the *HcCreb* gene plays a role in the process of melanin synthesis and nacre color formation in *H*. *cumingii*.

## 1. Introduction

*Hyriopsis cumingii* is a freshwater mussel that can produce high-quality freshwater pearls and is currently accounting for 95% of China’s pearl production [1]. Color, luster, size, thickness, shape, and surface defects are the six main aspects considered in the evaluation of pearl quality [2], and color is particularly important [3]. Previous studies have shown that pearl color is similar to the correspondent shell nacre color of the mantle tissue, which is determined by genetic factors [4]. Although existing research data do not provide a systematic and clear description of the process regulating nacre color formation, factors such as metal elements, porphyrins, carotenoids, melanin and physical structure may play an important role [5]. Jiang et al. [6] compared and analyzed the metal elements present in the mantle and interstitial fluid of *H*. *cumingii* of different colors and found that nacre color is correlated with the content of Fe, Mg, Co, and Mn. Zhang et al. [7] found that pearl color is mainly determined by porphyrin and metalloporphyrin, while Li et al [8] found that by adding carotenoids, the ability to accumulate carotenoids was higher in purple line mussels than in white line mussels. Shen [9] cloned a Mitf gene in *H*. *cumingii*, and found that the cloned gene, *HcMitf*, plays an important role in melanin synthesis, nacre formation and shell pigmentation.

Creb (Cyclic AMP response element binding protein) is a regulatory factor that controls cell proliferation, survival, differentiation and other processes by regulating the expression of a series of downstream genes. Creb is a member of the Creb/ATF family, which includes eight molecular subtypes, and it is a nuclear regulatory factor that regulates transcription through autophosphorylation [10,11]. Montrminy M R et, al. [12] first found and named cAMP response element binding protein, or “Creb”. During melanin production, cAMP binds to the regulatory subunits of PKA and induces the dissociation of the catalytic subunit from the holoenzyme complex. The released catalytic subunit is then activated and finally translocates to the nucleus and phosphorylates at Ser133 to activate the Creb, thus regulating the synthesis of melanin [13]. Among shellfish, Yu [14] found that Creb may be involved in the regulation of physiological processes in *Pteria Penguin*. Zhu et al. [15] cloned a Creb gene in *Crassostrea ariakensis*, and found that Creb may be involved in the regulation of physiological reactions connected to the immune response. Song [16] screened a Creb gene in *Pteria Penguin*, and found that it was distributed in different tissues, explaining the relationship between this gene and shell color based on the mRNA level.

In this study, a new Creb gene (*HcCreb*) was identified in *H*. *cumingii*, and its full length was cloned. The expression level of the *HcCreb* gene was detected in different tissues. *In situ* hybridization was used to detect the distribution of the level of mRNA expression in the mantle. After arbutin treatment, the expression of *HcCreb* decreased significantly. By further testing the changes of mantle melanin content, it was found that melanin content in the marginal membrane after arbutin treatment decreased significantly compared to the control group (*p*<0.01). These findings may help elucidate the role of *HcCreb* genes in the formation of nacre color in *H*. *cumingii*.

## 2. Materials and methods

### 2.1. Experimental materials

**Fig 1:**
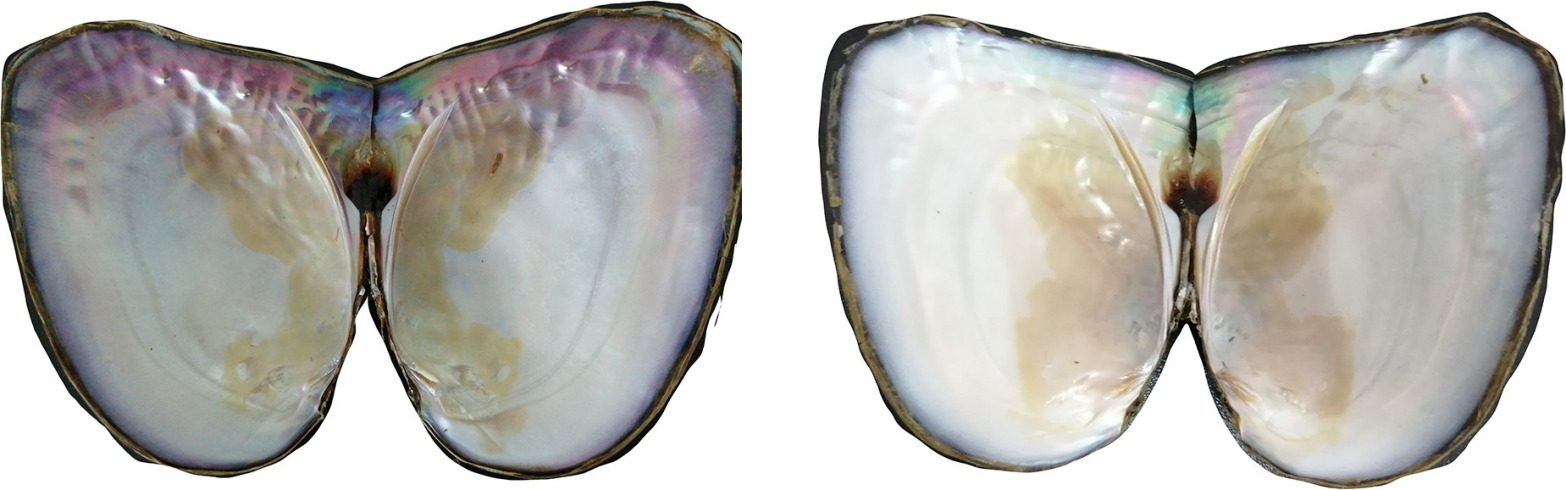
Purple (left) and white (right) *H*. *cumingii* mussels used in the experiment.

Two-year-old healthy *H*. *cumingii* mussels (with an average shell length of 10 cm) with purple and white inner-shell colors were obtained from Xuan Cheng Farm of Zhexing Pearl Trading Co. Ltd., Anhui Province, China (Fig 1). Before the experiment, the mussels were placed in a laboratory water tank for oxygenation for about a week, and then fresh mantle samples were taken and stored at -80°C for later use.

### 2.2. Experimental method

#### 2.2.1. Total RNA extraction and cloning of the full-length *HcCreb*

The TRIzol method was used to extract total RNA from healthy mantle tissue samples. The SMARTer RACE 5’/3’ kit was used to synthesize RACE-Ready cDNA as a gene cloning template. The Creb gene fragment was obtained from *H*.*cumingii*’s mantle transcriptome library [17] (Table 1), and the specific primers were designed by Primer 5.0 in order to perform PCR amplification and verify the sequence. According to the SMARTer RACE 5’/3’ kit instructions, respectively, designing 5’-RACE and 3’-RACE specific primers, performing RACE cloning, and performing sequence sequencing by Sangon(Shanghai, China) were used to obtain the full length of Creb gene.

**Table 1:** Primers used in the study.

Primer name	Sequence (5’–3’)	Purpose
HcCreb-F	GATGCTCCTTGCTTGTTAGATG	Partial fragment amplifification of HcCreb
HcCreb-R	CACACCAGCCGTTTGTTGAG	Partial fragment amplifification of HcCreb
HcCreb-3’	GATGCTCCTTGCTTGTTAGATG	3’RACE
HcCreb-5’	CATCTAACAAGCAAGGAGCATC	5’RACE
HcCreb-RT-F	GACTGTGTGCTGCTCAACAA	qPCR
HcCreb-RT-R	CTTGCACACTGAGCACAGAA	qPCR
HcCreb-Y-F	GACTGTGTGCTGCTCAACAA	*In situ* hybridization
HcCreb-Y-R	TAATACGACTCACTATAGGGCTTGCACACTGAGCACAGAA	*In situ* hybridization
EF1α-F	GGAACTTCCCAGGCAGACTGTGC	qPCR internal control
EF1α-R	TCAAAACGGGCCGCAGAGAAT	qPCR internal control
HcTyr-RT-F	TCGACAGCTGGGTGTACATT	qPCR
HcTyr-RT-R	GCACTTTGCCGTCTTGAATA	qPCR
HcMitf-RT-F	TCAACAGGAGGCCTGCCATA	qPCR
HcMitf-RT-R	TGTGTTGCGGACAGGAAGTG	qPCR

#### 2.2.2.Sequence analysis

The ORF Finder program was used (https://www.ncbi.nlm.nih.gov/orffinder/) to predict the open reading frame(ORF) and coding amino acid sequences of the *HcCreb* gene [18]. Smart Blast was used to analyze the amino acid sequence homology and Simple Modular Architecture Research Tool SMART software (http://smart.embl-heidelberg.de/) to identify protein domains [19]. The amino acid sequences, functional sites, molecular weight and isoelectric point of *HcCreb* were predicted using ExPASy (http://web.ex pasy.org/) [20]. Clustalx software was used for multiple sequence alignment analysis [21] and MEGA 5.2 (Arizona State University, USA) to construct a phylogenetic tree [22].

#### 2.2.3.Tissue expression analysis

Mantle, adductor muscle, gill, foot and hepatopancreas samples were taken from six healthy *H*. *cumingii* individuals and were used for RNA extraction. The RNA was then reverse-transcribed to cDNA by using the SYBR^®^Premix Ex Taq™ II (Tli RNaseH Plus) (TaKaRa). Bio-Rad-CFX-96 (Bio-Rad, USA) was used for fluorescence quantitative PCR. The PCR reaction mixture was as follows: SYBR^®^Premix Ex Taq™ II (Tli RNaseH Plus), 10 μL; upstream and downstream primers, 0.8 μL; ddH_2_O, 6.8 μL and cDNA template, 1.6 μL. The reaction was repeated three times. The reaction cycles were set as follows: initial denaturation at 95°C for 30 s; 95°C for 5 s, 56°C for 35 s, 40 cycles; 72°C for 30 s. Based on previous research results at our laboratory, EF-1α was used as the internal reference [23] (Table 1).

#### 2.2.4. *In situ* hybridization

Specific primers were designed and the T7 promoter sequence-TAATACGACTCACTATAGGG was added to the 5’ end of the reverse primer (Table 1). The target fragment was obtained through PCR amplification and product purification, and in vitro transcription was carried out using the TransGen Biotech transcription kit. The fresh mantle tissue removed from a purple *H*.*cumingii* individual was put it in 4% paraformaldehyde for fixation and dehydration for 4 hours (in a refrigerator at 4°C), then it was put in 25% sucrose solution at 4°C overnight. The tissue was sliced to sections of 10–15 μm in thickness using a freezing microtome, and it was stored at -80°C. Subsequently, *in situ* hybridization was performed.

#### 2.2.5.Expression of the Creb gene in the mantle of *H*. *cumingii* after arbutin treatment

*H*. *cumingii* individuals were divided into three groups of 15 mussels each: one control group and two experimental groups treated with arbutin with different concentrations of 10 mM and 50 mM. Tissue samples were collected at 6 h, 12 h, 24 h and 48 h in each group. In the control group, *H*. *cumingii* was cultured in fresh water while in the experimental group individuals were kept at two different concentrations of arbutin-enriched freshwater. During the period in the tank they were fed a small amount of chlorella. After 6 h, 12 h, 24 h and 48 h, samples were taken from different tissues. RNA was extracted and reverse transcribed into the first strand cDNA. Then, the expression of Creb, Tyr(Tyrosinase) and MITF(Microphthalmia-associated transcription factor) genes in the mantle was analyzed through real-time PCR. The PCR protocol used was as follows: initial denaturation for 4 min at 94°C; 30 cycles at 94°C for 30 s, 55°C for 30 s, and 72°C for 1 min, and a fifinal elongation step at 72°C for 5 min.

#### 2.2.6. Melanin assay

In this experiment, the concentration of 50 mM, time point for 6h as the experimental group. In this experiment, we obtained mantle tissues from six mussels in the experimental group and the blank group, respectively. Then mix them separately as one sample. Three technical replicates were performed for each sample. The melanin content present in the mantle tissue of *H*.*cumingii* cultured with arbutin was determined using the Tissue Melanin Assay Kit (Shanghai Haling Biotechnology Co., Ltd.). Take 500 mg mantle tissue and grind it in liquid nitrogen, then add pyrolysis liquid. Supernatants were discarded following centrifugation and the treatment solution was added. Samples were then centrifuged and the supernatants were discarded. Precipitates were then dissolved in alkaline solution. Samples were mixed with the buffer and incubated at 60°C for 30 min. AD2000 spectrophotometer (wavelength: 360 nm) was used to obtain the absorbance reading. Melanin content of experimental samples were measured based on the standard curve prepared by the standard products (0–80μ g/ml) provided in the kit.

#### 2.2.7 Statistical analysis

Data are shown as the mean ± SD and was analysed using SPSS 17.0 software. Differences were recognized as signifificant when *p* < 0.05 and highly signifificant when *p* < 0.01.

## 3. Results

### 3.1. The cDNA cloning and sequence analyses of *HcCreb*

The full length of the *HcCreb* (GenBank accession No.MT816340) gene was obtained by 3’ and 5’ RACE cloning and through the publicly available sequences. The *HcCreb* gene sequence is 1463 bp in total, of which the 5’-UTR was 15 bp, the 3’-UTR was 344 bp, and the ORF was 1104 bp long, encoding a total of 367 amino acids. The molecular weight of mature protein content corresponding to the amino acid sequences was 119.63 kDa. The isoelectric point calculated was 4.43.

### 3.2. Phylogenetic analysis

**Fig 2:**
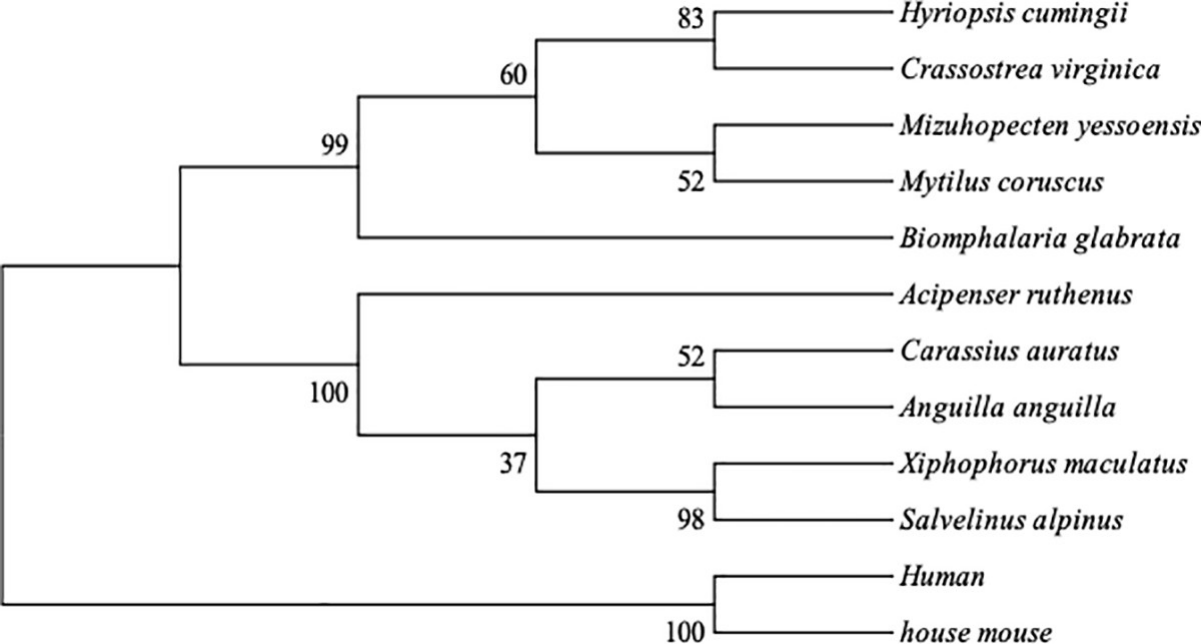
Phylogenetic analysis based on multiple sequence alignment.

Using Mega 5.2 software, the Creb gene of *H*.*cumingii* was sequenced and compared with the Creb gene of other species in order to construct a phylogenetic tree. As shown in Fig 2, *HcCreb* is located close to the Creb of *Crassostrea virginica* with a confidence level of 83%.

### 3.3.Tissue expression analysis

**Fig 3:**
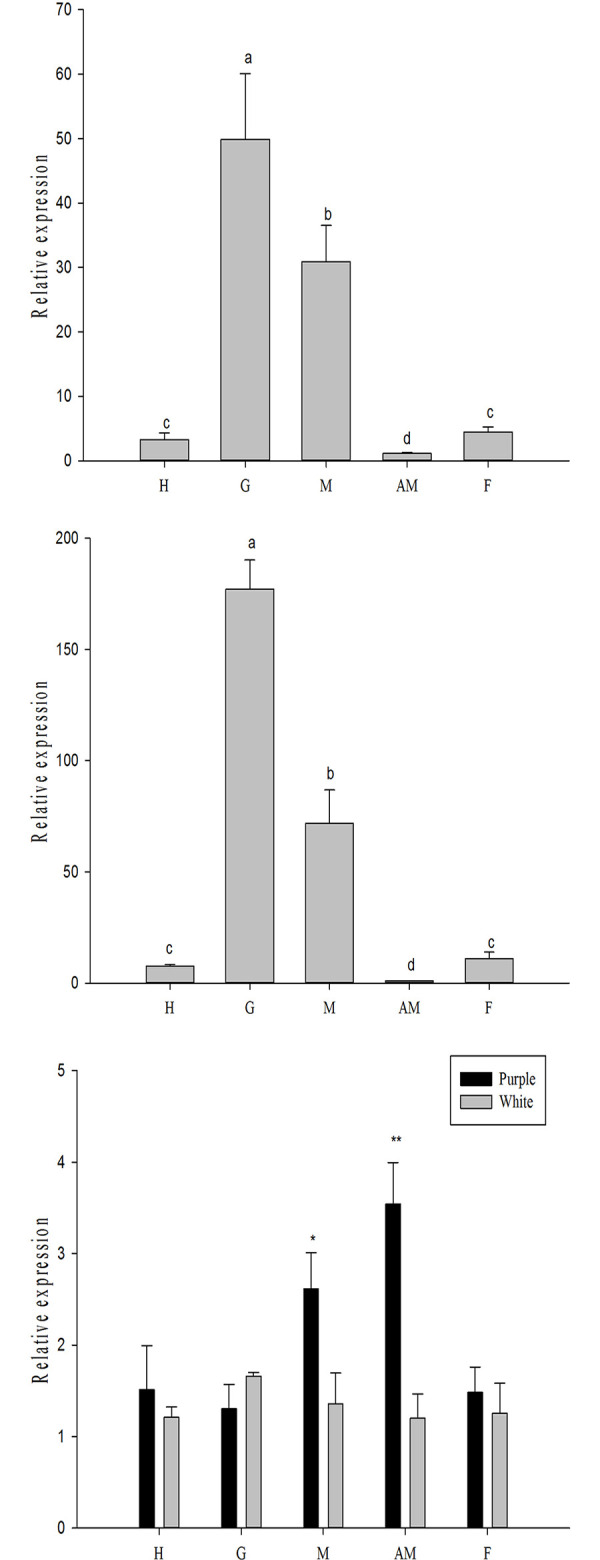
Relative expression level of Creb. The relative expression level of Creb in various tissues of purple (A) and white (B) mussels. Comparison of Creb expression in white and purple mussels (C). H: hepatopancreas, G: gill, M: mantle, AM: adductor muscle, F: foot. Data from the qPCR experiments are expressed as the means ± SD (n = 6). Bars with different letters indicate significant differences (p < 0.05).

The relative expression of the *HcCreb* gene in the tissues of white and purple *H*.*cumingii* was detected through qPCR. The results in Fig 3 show that *HcCreb* was expressed in all tissues of both white and purple mussels with a significant difference in expression between the adductor muscle (*p*<0.01) and mantle (*p*<0.05) tissue. The other tissues do not show significant differences. Except for gill tissue, the level of mRNA expression of the other tissues in purple mussels was higher than that in white mussels. In gill tissue, the level of mRNA expression was the highest for both white and purple mussels, followed by that in the mantle.

### 3.4. *In situ* hybridization results

**Fig 4:**
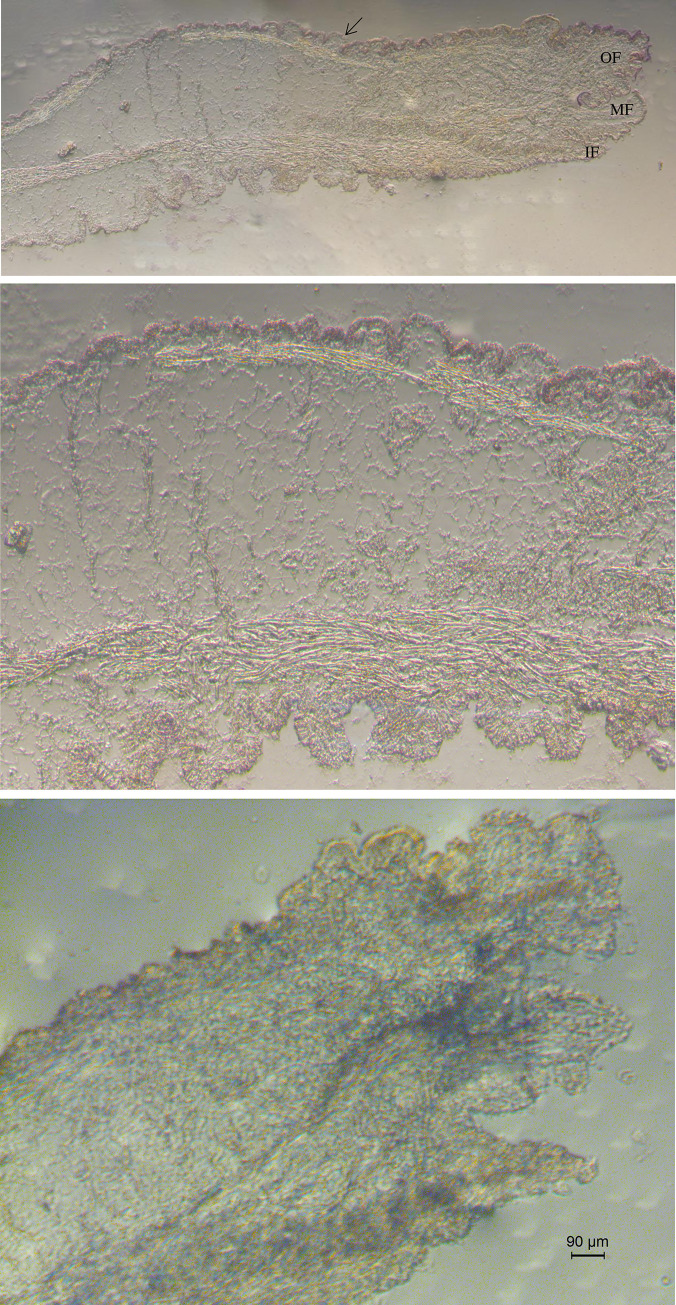
*In situ* hybridization analysis of *HcCreb*(A) in the mantle. B was higher magnifications of A, C was background. IF, inner fold; MF, middle fold; OF, outer fold. Data from the qPCR experiments are expressed as the means ± SD (n = 6). Bars with different letters indicate significant differences (p < 0.05).

The specific expression position of the *HcCreb* gene in the mantle tissue was determined by *in situ* hybridization. The results are shown in Fig 4, where it is visible that the positive hybridization signal mainly appeared in the epithelial cells of the dorsal membrane on the outer fold of the mantle (arrow), and there was no obvious signal in other positions. No positive signal was detected in the negative control.

### 3.5. Expression analysis of the Creb gene in the mantle tissue after arbutin treatment

**Fig 5:**
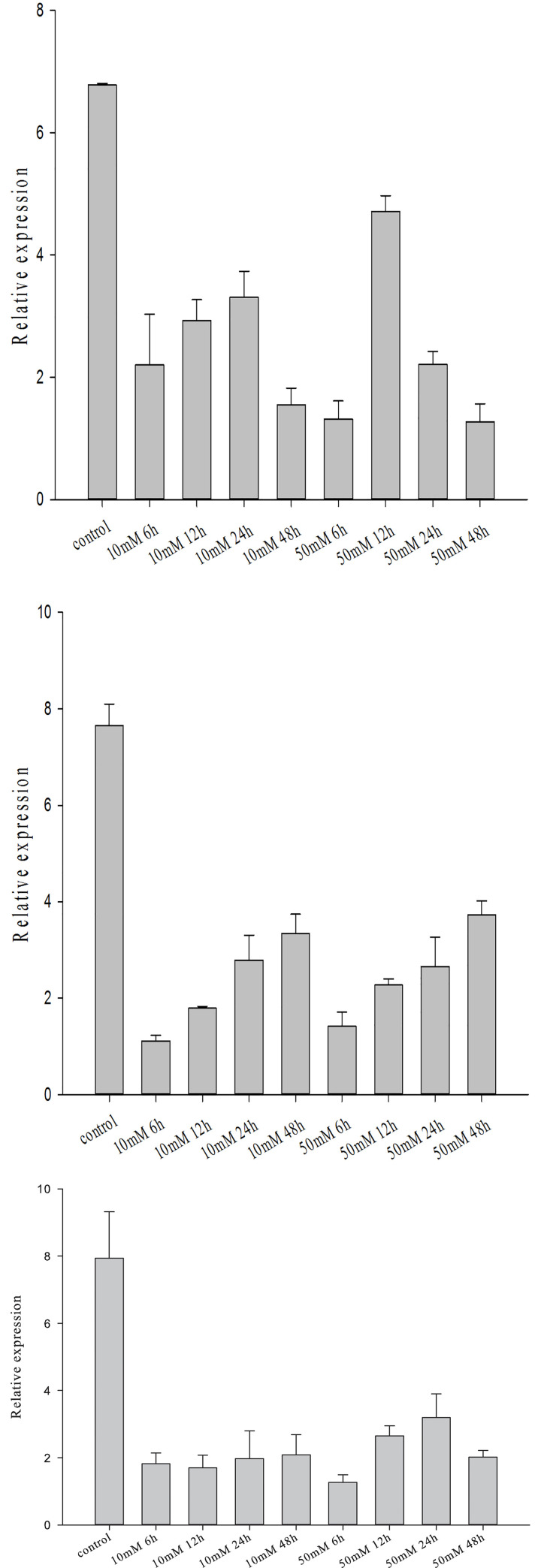
Expression levels of *HcTyr* (A), *HcMitf* (B), *HcCreb* (C) genes in the mantle of *H*.*cumingii* after arbutin treatment. Data from the qPCR experiments are expressed as the means ± SD (n = 6). Bars with different letters indicate significant differences (p < 0.05).

The expression levels of Tyr, Mitf and Creb genes in the marginal membrane of *H*.*cumingii* treated with arbutin at concentrations of 10 mM and 50 mM at 6 h, 12 h, 24 h and 48 h, are shown in Fig 5. After 6 h of arbutin treatment at two different concentrations, the mRNA expression of *HcTyr* (*H*.*cumingii* Tyrosinase gene), *HcMitf* (Microphthalmia-associated transcription factor of *H*.*cumingii*), and *HcCreb* decreased significantly compared to the control group (p<0.01). After 12 h of arbutin treatment at two different concentrations, the expression levels of *HcTyr*, *HcMitf* and *HcCreb* increased compared to the levels seen after 6 hours, which may be due to the stress response of *H*. *cumingii*.

### 3.6. Detection of melanin content in the mantle

**Fig 6:**
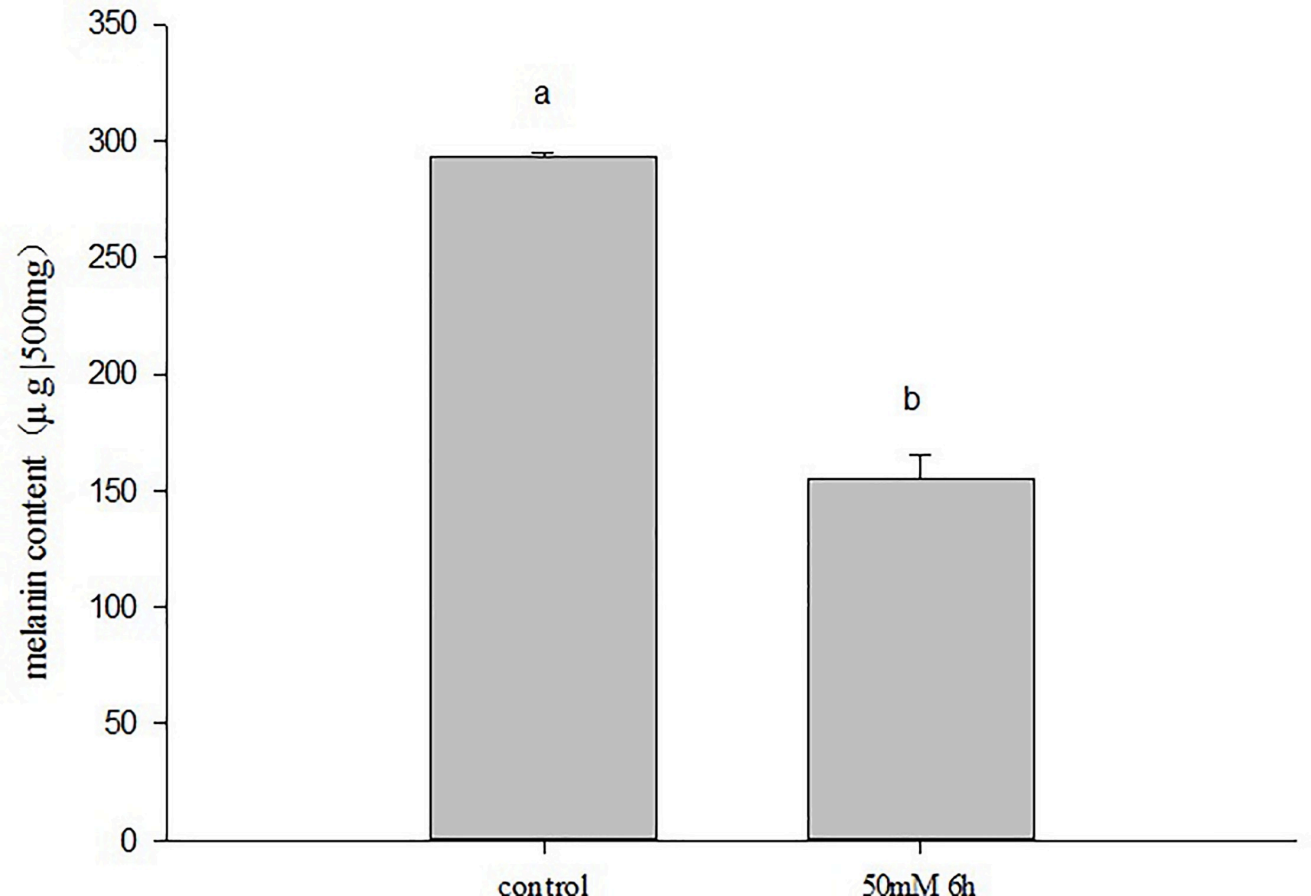
Melanin content in mantle tissue.

As the expression levels of H*cCreb*, H*cMitf*, and *HcTyr* changed significantly after arbutin treatments, changes in melanin content were also detected in the mantle (Fig 6). Results show that the melanin content in the mantle after arbutin treatment was significantly lower than that of the control group, which was not exposed to any treatment (p <0.05), melanin content was signifificantly reduced by 47.1% when compared with the blank group.

## 4. Discussion

In this study, a *HcCreb* gene was fully cloned and the role of *HcCreb* in the synthesis of melanin and color formation of the nacre in *H*.*Cumingii*, was investigated for the fist time. Through the analysis of Except for gill tissue, the level of mRNA expression of the other tissues in purple mussels was higher than that in white mussels it was found that *HcCreb* is expressed in the mantle, adductor muscle, gills, foot, and hepatopancreas of *H*.*cumingii*, and that the expression level is higher in the gill and mantle. The specific expression position of the *HcCreb* gene in the mantle tissue was determined by *in situ* hybridization. Strong and specific positive hybridization signals were detected in the dorsal epithelial cells of the mantle pallial layer, indicating that *HcCreb* may be involved in nacre formation. By further comparing these results with except for gill tissue, expression level of the other tissues in purple mussels was higher than that in white mussels levels in other tissues, it is concluded that *HcCreb* was specifically involved in the formation of purple nacre. Arbutin is a natural active substance that is generally extracted from a variety of different plants [24]. It is a glucosylated hydroquinone derivative. It belongs to hydroquinone glucosides and has specific physiological functions. It is widely found in animals, plants and microbial cells. Arbutin can effectively inhibit the activity of biological tyrosinase in the skin, block the formation of melanin [25], and accelerate the decomposition and excretion of melanin through direct binding with tyrosinase [26]. Arbutin has an inhibitory effect on melanin synthesis in organisms, therefore it was used in the experiments on *H*. *cumingii*. After arbutin treatment, the expression of *HcTyr*, *HcMitf*, and *HcCreb* genes decreased significantly compared to the control group. Creb binds to cAMP and autophosphorylation. The transcription regulation of Mitf by Creb in melanin formation is affected by cAMP concentration. Some studies have shown that arbutin and other melanin inhibitors can inhibit the formation of melanin by reducing the level of cAMP and down regulating the expression of melanin related proteins (such as Creb) [27,28]. Therefore, the expression of Creb gene decreased significantly after arbutin treatment. Arbutin not only affects cAMP, but also combines with tyrosinase to affect its enzyme activity, thus affecting its gene expression. Tyrosinase is a key enzyme for melanin synthesis. Chen et al. [29] found that the Tyr gene in H.cumingii is also involved in nacre formation and that, by regulating the synthesis of melanin, it may affect nacre color. Marin [30] suggested that the Tyr gene is mainly related to the color formation of bivalve shells and nacre. Nagai et al. [31] cloned two tyrosinase genes in *Pinctada martensii*, and found that they were specifically expressed in the mantle. Arbutin competitively and reversibly inhibits tyrosine, therefore blocking the synthesis of dopa and dopaquinone, and inhibiting the production of melanin. By further testing the melanin content in the mantle tissue after arbutin treatment, it was found that it decreased significantly compared to the control group suggesting that *HcCreb* may play a role in the synthesis of melanin. These results indicate that *HcCreb* gene may affect nacre color formation by participating in melanin synthesis. However, the specific mechanism of *HcCreb* gene is still unclear due to the preliminary research.

In summary, this study for the first time indicated that *HcCreb* may be an important factor in the synthesis of melanin ultimately affecting the formation of nacre color. These findings can contribute to the understanding of the processes determining pearl color, and thus improve the pearl production process.
